# A Collagen-Derived Peptide Fraction CCAP1 from *Colla Corii Asini* Attenuates UVB-Induced Skin Photoaging via an AMPK-Dependent Nrf2/NF-κB Axis

**DOI:** 10.3390/molecules31213964

**Published:** 2026-11-03

**Authors:** Fen Du, Zhuang Miao, Wanxiu Cao, Yangen Sun, Xiaomei Feng, Haibin Liu, Changwei Wang, Jie Cheng, Xueyuan Fu, Dongwei Wang, Bafang Li, Xiaobo Duan, Chuyi Liu

**Affiliations:** 1 Marine Biomedical Research Institute of Qingdao, Ocean University of China, Qingdao 266100, China; dufen0402@ouc.edu.cn (F.D.);; 2 Shandong Key Laboratory of Gelatine Medicines Research and Development, Dong’e Ejiao Co., Ltd., Liaocheng 252200, China; miaozhuang12@dongeejiao.com (Z.M.);

**Keywords:** skin photoaging, collagen-derived peptides, AMPK signaling, untargeted metabolomics, UVB irradiation, oral supplementation

## Abstract

Chronic UVB exposure drives extrinsic skin aging through oxidative stress, inflammation, and collagen degradation. Although oral collagen peptides are promising photoprotective agents, most preparations lack defined composition and mechanistic validation. Here, we characterized CCAP1, a type I collagen-derived oligopeptide fraction (~1.7 kDa; 94.69% collagen-related components) prepared from *Colla Corii Asini* by dual-enzymatic hydrolysis and membrane fractionation. In UVB-irradiated mice, oral CCAP1 (100 and 200 mg/kg/d, 28 days) markedly alleviated UVB-induced epidermal hyperplasia, restored skin hydration and dermal collagen types I/III/IV, and suppressed MMP1. Untargeted skin metabolomics nominated AMPK signaling as the central node; Western blotting confirmed that CCAP1 activated the AMPK/Nrf2 axis while suppressing NF-κB phosphorylation, with elevated SOD activity and reduced MDA content. In UVB-injured HaCaT keratinocytes, CCAP1 restored cell viability and reduced intracellular ROS, and both effects were abolished by the AMPK inhibitor Compound C, establishing AMPK as a functionally required mediator. Collectively, CCAP1 is a composition-defined, orally bioavailable collagen peptide fraction that attenuates UVB-induced photoaging via an AMPK-dependent Nrf2/NF-κB regulatory program.

## 1. Introduction

Skin photoaging is a major form of extrinsic skin aging, typically caused by chronic UVB exposure, and is characterized by wrinkles, erythema, epidermal hyperplasia, and progressive loss of dermal collagen [1,2]. At the molecular level, UVB irradiation initiates a self-amplifying cascade in the skin. Excessive reactive oxygen species (ROS) production depletes endogenous antioxidant defenses and activates the pro-inflammatory transcription factor NF-κB, which in turn up-regulates matrix metalloproteinases (MMPs), leading to the degradation of collagens, particularly types I, III, and IV, and disruption of the dermal extracellular matrix [3,4]. Notably, this cascade is not merely linear. ROS-driven NF-κB activation promotes the release of pro-inflammatory cytokines such as TNF-α and IL-6, which in turn amplify ROS generation [5]. This creates a self-amplifying loop that perpetuates oxidative stress and drives MMP-mediated matrix degradation [6,7].

Breaking this cycle requires an upstream target capable of simultaneously suppressing oxidative stress and inflammatory signaling. AMPK, a cellular energy sensor that integrates stress signals, is an attractive candidate in this regard. Upon activation, AMPK promotes Nrf2 nuclear translocation and antioxidant enzyme transcription while suppressing NF-κB-driven inflammatory signaling [8,9]. Consistent with this, pharmacological or nutritional activation of the AMPK/Nrf2 axis has been shown to alleviate UVB-induced photodamage in keratinocytes and dermal fibroblasts, whereas AMPK inhibition abolishes these protective effects, indicating that AMPK is a functionally required upstream regulator [3,10]. Notably, AMPK phosphorylation is suppressed in chronically UVB-irradiated mouse skin in vivo, establishing AMPK down-regulation as an early event in photoaging [11]. However, in research on collagen peptide-based anti-photoaging agents, whether AMPK truly dominates this process has not been systematically examined through an unbiased discovery approach. Most studies use pathway-targeted designs and assess only preselected endpoints such as MAPK/AP-1 or TGF-β/Smad, making it impossible to determine which upstream node actually drives the observed protection [12,13].

This gap is particularly relevant to oral collagen peptides, which bypass the stratum corneum barrier and provide systemic skin protection [14,15,16]. Colla Corii Asini (CCA), a traditional medicinal and edible gelatin prepared from donkey hide, is rich in collagen-derived peptides and has long been valued for its skin-nourishing properties [17,18]. However, research on CCA-derived peptides remains fragmented. Many extraction and purification protocols are crude, yielding peptide mixtures with poorly defined protein sources, broad molecular weight distributions, and inconsistent bioactivity [19]. Mechanistically, CCA-derived peptides include both collagen-derived and non-collagen-derived sequences, and their activities have been explored separately. For instance, Ma et al. reported that non-collagenous HSP90AA1-derived peptides from CCA promote collagen regeneration in photoaged skin via the TGF-β/Smad pathway [20,21]. However, whether collagen-derived peptide fractions from CCA can act through other upstream nodes, such as AMPK, to protect against photoaging remains unclear. Moreover, few studies have linked oral absorption to cutaneous mechanisms through an integrated chain of evidence that combines unbiased discovery with pharmacological causal validation. Thus, whether a defined CCA-derived collagen peptide fraction can be absorbed orally and act through a dominant upstream axis, such as AMPK, to protect against photoaging remains an open question.

To address these gaps, we prepared a defined collagen-derived oligopeptide fraction (CCAP1) from CCA by dual-enzymatic hydrolysis combined with membrane fractionation, and characterized its compositional features. The oral anti-photoaging efficacy of CCAP1 was then evaluated in vivo. Mechanistically, untargeted skin metabolomics was first used to nominate the upstream regulatory pathway; the nominated pathway was further validated in mouse skin by Western blotting, and its causal necessity was examined in UVB-irradiated HaCaT keratinocytes using pharmacological AMPK inhibition. This study provides a well-characterized, orally bioavailable CCA-derived collagen peptide fraction for skin photoprotection, and offers new mechanistic insight into AMPK-centered photoprotection by food-derived collagen peptides, supporting the traditional skin-nourishing use of CCA.

## 2. Results

### 2.1. Preparation, Compositional Characterization, and In Vitro Antioxidant Activity of CCAP1

CCAP1 was prepared from *Colla Corii Asini* (CCA) by activity-guided dual enzymatic hydrolysis and subsequent membrane fractionation. The lyophilized product exhibited a protein content of 98.21% on a dry basis, determined using the Kjeldahl method. Peptidomic profiling by UHPLC Q Orbitrap MS demonstrated that collagen-related proteins dominated the composition of CCAP1, accounting for 94.69% of the total relative protein abundance. The four most abundant proteins, COL1A1, COL1A2, COL3A1 and COL5A2, together accounted for 84.43% of total abundance, while peptides assigned to the type I collagen α-chains COL1A1 and COL1A2 represented 75.20% (Table 1). A complete list of all identified proteins is provided in Table S1. These results indicate that CCAP1 is predominantly composed of type I collagen-derived peptides.

At the peptide level, GPC analysis showed an apparent weight-average molecular weight of ~1.7 kDa, consistent with extensive hydrolysis of high-molecular-weight gelatin components in CCA into low-molecular-weight oligopeptides. The ten most abundant oligopeptides were identified via MS/MS (Table 2, Figure S1); one pair of isomeric peptides coeluted with identical *m*/*z* and retention time and was not chromatographically resolved. P(+15.99) indicates proline hydroxylation, a post-translational modification that yields hydroxyproline (Hyp), corresponding to a mass increment of ~16 Da. Due to their identical monoisotopic mass and co-elution, Leu/Ile discrimination could not be achieved using diagnostic fragment ions from the acquired MS/MS spectra; therefore, these two sequences are tentatively assigned as isomeric candidates. The identified peptides were enriched in Gly-X-Y motifs and predominantly mapped to collagen α-chains (Table S1). Among the top 10 peptides, 80% contained hydroxyproline (Hyp) residues and 60% contained at least one canonical Gly-X-Hyp motif.

Consistent with this, the Hyp content of CCAP1 was 10.38% (*w*/*w*, dry basis), supporting its collagen-derived nature and enrichment in Hyp-containing sequences. GPC analysis further showed that 80.77% of the CCA peak area corresponded to apparent molecular weights > 3 kDa, whereas 83.65% of the CCAP1 peak area was <3 kDa (Figure S2). Under the same assay conditions (200 μg/mL), CCAP1 scavenged 79.28% of superoxide radicals, whereas CCA scavenged 32.46% (*p* < 0.0001). Collectively, these results indicate that CCAP1 is a CCA-derived collagen oligopeptide mixture with well-characterized major components.

### 2.2. Intestinal Absorption and Oral Safety of CCAP1

**Figure 1:**
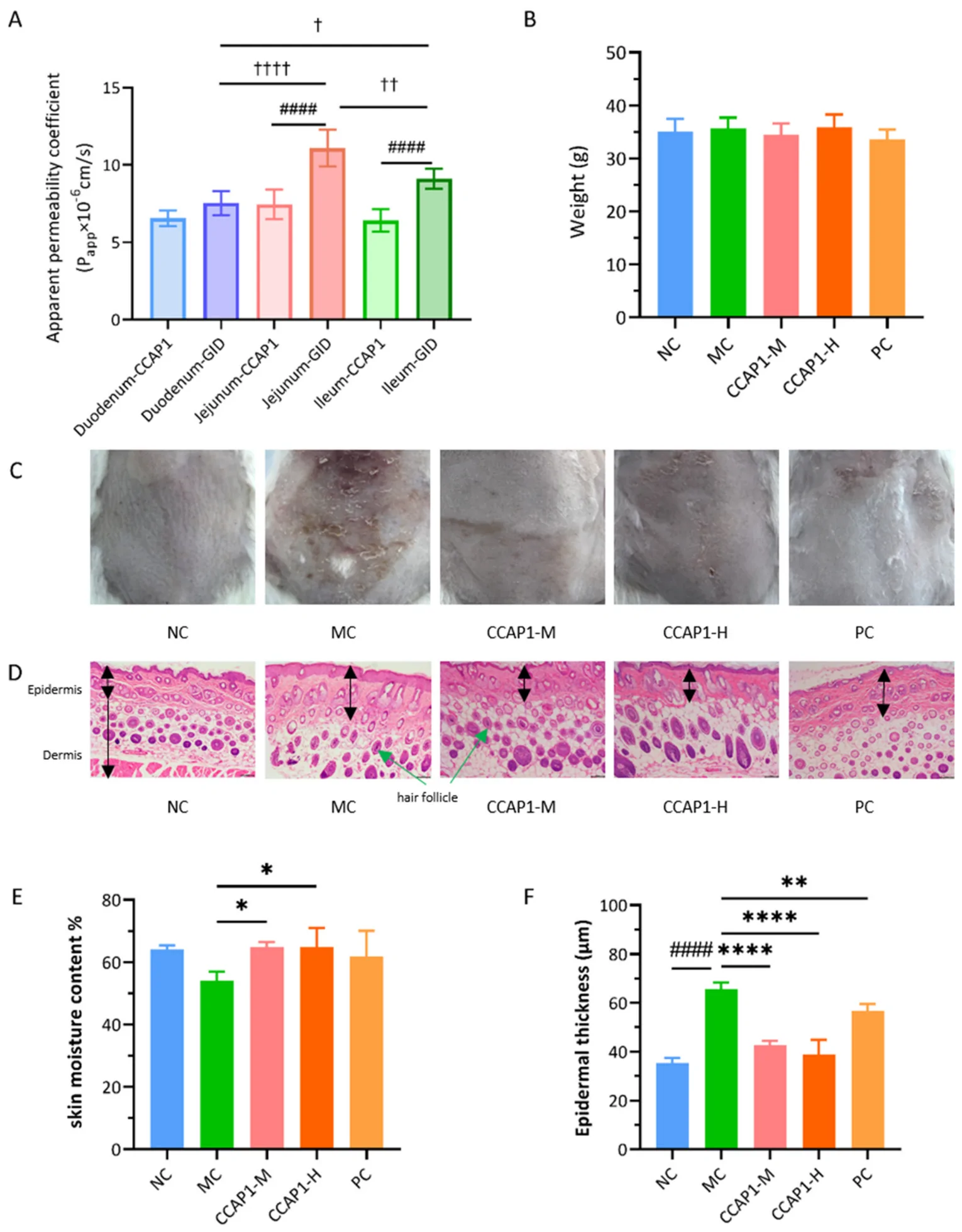
Intestinal absorption, oral safety, and anti-photoaging effects of CCAP1 in mice. (**A**) Apparent permeability coefficient (*P_app_*, ×10^−6^ cm/s) of CCAP1 across duodenal, jejunal, and ileal segments, with or without simulated gastrointestinal digestion (GID). Data are presented as mean ± SD (*n* = 6). ^####^ *p* < 0.0001 vs. undigested CCAP1 in the same intestinal segment; ^†^ *p* < 0.05, ^††^ *p* < 0.01, ^††††^ *p* < 0.0001 for comparisons among duodenal, jejunal and ileal segments within the GID group. (**B**) Body weight after the 28-day oral intervention; no significant differences were observed among the five groups. (**C**) Representative macroscopic photographs of mouse dorsal skin from each group. (**D**) Hematoxylin–eosin (H&E)-stained skin sections; (**E**) skin surface moisture content (%). (**F**) Epidermal thickness (μm). Data are presented as mean ± SD (*n* = 10). ^####^ *p* < 0.0001 vs. NC; * *p* < 0.05, ** *p* < 0.01, **** *p* < 0.0001 vs. MC. NC, normal control; MC, UVB-irradiated model control; CCAP1-M, 100 mg/kg/day; CCAP1-H, 200 mg/kg/day; PC, ergothioneine positive control (100 mg/kg/day).

To determine whether CCAP1 can be absorbed after oral administration, the everted gut sac model was used to measure its apparent permeability coefficient (*P_app_*) across three major intestinal segments in rats (Figure 1A). After simulated gastrointestinal digestion (GID), CCAP1 exhibited the highest *P_app_* in the jejunum, significantly exceeding the values in the duodenum and ileum. Moreover, GID markedly enhanced CCAP1 permeability in all three segments compared with the undigested form (*p* < 0.0001), indicating that gastrointestinal digestion is a critical prerequisite for its efficient intestinal uptake.

Having confirmed intestinal absorption, the safety of 28-day oral administration of CCAP1 was further evaluated. Under the pathological conditions of repeated UVB irradiation, 28-day oral CCAP1 did not affect body weight in any group (Figure 1B), and the organ indices remained comparable among the five groups (Figure S3). These results indicate that CCAP1 possesses favorable jejunal absorption and good short-term oral tolerability. The BCA assay quantifies total peptide-bond-containing substances and cannot discriminate intact bioactive oligopeptides, digestive fragments or free amino acids. Accordingly, the calculated apparent permeability coefficient reflects total permeated nitrogenous material rather than individual intact bioactive peptides. Future pharmacokinetic detection using LC-MS/MS or isotope-labelled signature peptides will help identify which specific peptides cross the intestinal epithelium intact.

### 2.3. Ameliorative Effect of CCAP1 on UVB-Induced Macroscopic and Histological Skin Photodamage

Building on its favorable absorption and safety profile, the anti-photoaging efficacy of orally administered CCAP1 was further evaluated. Macroscopically, the NC group displayed smooth skin without erythema, scaling, or wrinkling, whereas chronic UVB exposure induced severe erythema, desquamation, and a rough, wrinkled appearance in the MC group (Figure 1C). Oral CCAP1 markedly attenuated these macroscopic alterations, with the CCAP1-H group closely resembling the ergothioneine positive control.

Histological examination and quantitative analysis (Figure 1D–F) revealed pronounced epidermal hyperplasia with inflammatory cell infiltration in the MC group; relative to the NC group, epidermal thickness was significantly increased (*p* < 0.0001). Both doses of CCAP1 significantly suppressed this hyperplastic response (*p* < 0.0001) and significantly increased skin moisture content in photoaged skin (*p* < 0.05). Collectively, oral CCAP1 significantly alleviated UVB-induced skin photoaging by ameliorating macroscopic lesions, suppressing epidermal hyperplasia, and restoring skin barrier function.

### 2.4. Preservation of Dermal Collagen Integrity and Suppression of MMP1 by CCAP1

**Figure 2:**
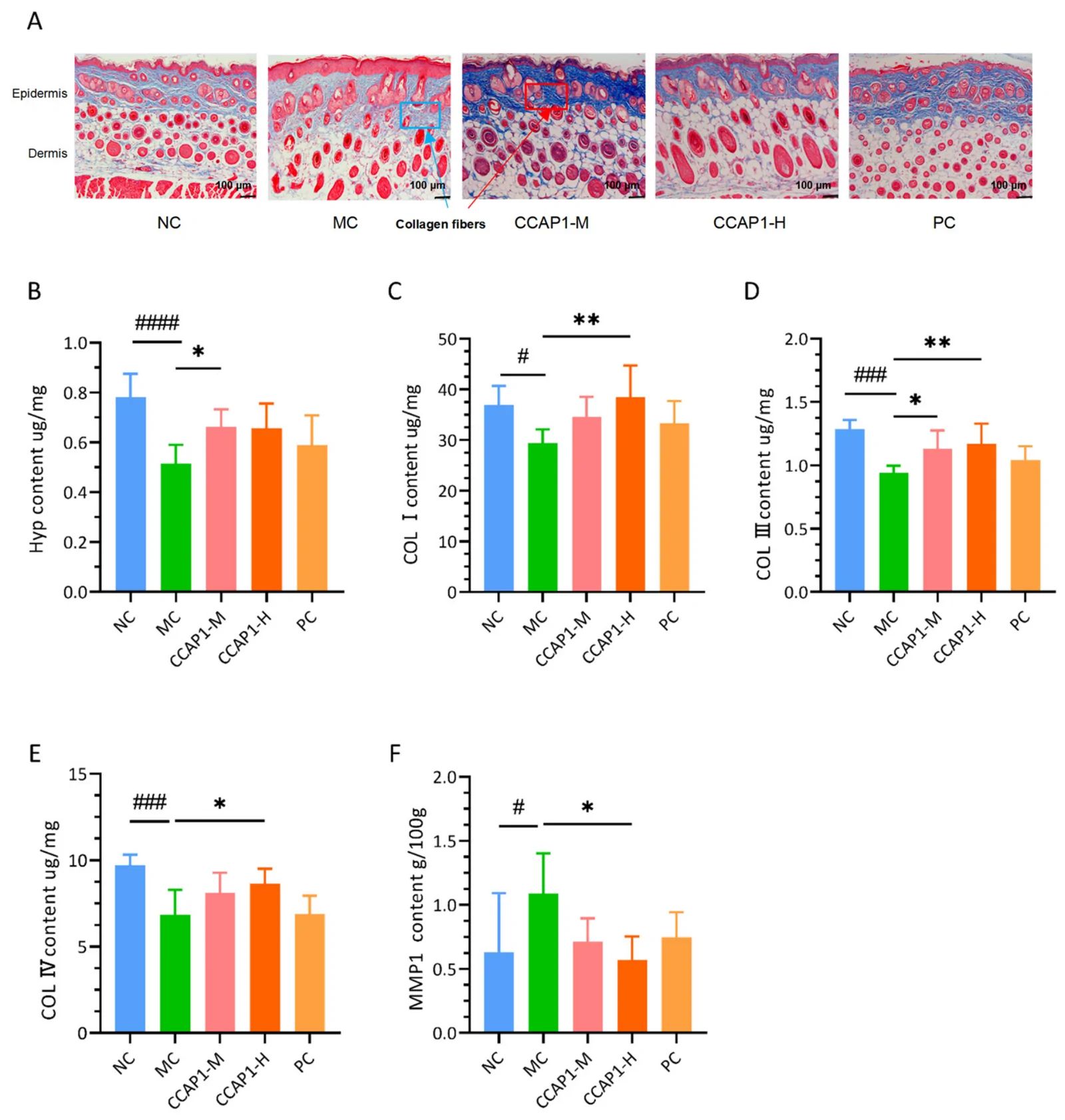
CCAP1 preserves dermal collagen and suppresses MMP1 in UVB-exposed mouse skin. (**A**) Masson’s trichrome staining of skin sections showing dermal collagen fiber distribution; scale bar = 50 μm (*n* = 10). (**B**) Hydroxyproline (Hyp) content in skin homogenates (*n* = 10). (**C**–**E**) Contents of collagen type I (COL I), type III (COL III), and type IV (COL IV) (*n* = 10). (**F**) MMP1 content (*n* = 10). Data are presented as mean ± SD. ^#^ *p* < 0.05, ^###^ *p* < 0.001, ^####^ *p* < 0.0001 vs. NC; * *p* < 0.05, ** *p* < 0.01. NC, normal control; MC, UVB-irradiated model control; CCAP1-M, 100 mg/kg/day; CCAP1-H, 200 mg/kg/day; PC, ergothioneine positive control (100 mg/kg/day).

Based on the above improvements in skin phenotype, the protective effect of CCAP1 on the dermal collagen matrix was further evaluated. Masson staining showed that chronic UVB exposure caused marked loss and fragmentation of dermal collagen fibers, whereas the oral CCAP1 groups maintained a denser and better-organized collagen network relative to the MC group, with the CCAP1-H group resembling the positive control (Figure 2A). Consistently, UVB irradiation significantly reduced skin hydroxyproline (Hyp) content, which was restored by high-dose CCAP1 (Figure 2B). Quantitative ELISA (Figure 2C–E) further revealed that, relative to the NC group, UVB irradiation significantly reduced the contents of type I collagen (*p* < 0.05), type III collagen (*p* < 0.001), and type IV collagen (*p* < 0.001); high-dose CCAP1 restored all three collagen subtypes toward normal levels (type I, *p* < 0.01; type III, *p* < 0.01; type IV, *p* < 0.05). Conversely, the key collagen-degrading enzyme MMP1 was markedly up-regulated in the MC group (*p* < 0.05) and significantly suppressed by CCAP1-H treatment (*p* < 0.05). Likewise, MMP2 and MMP9 exhibited a similar elevation under UVB exposure and were down-regulated by CCAP1-H (Figure S4). These results demonstrate that oral CCAP1 protects the dermal extracellular matrix by maintaining collagen abundance and suppressing MMP1-mediated degradation.

### 2.5. Untargeted Skin Metabolomics of CCAP1

**Figure 3:**
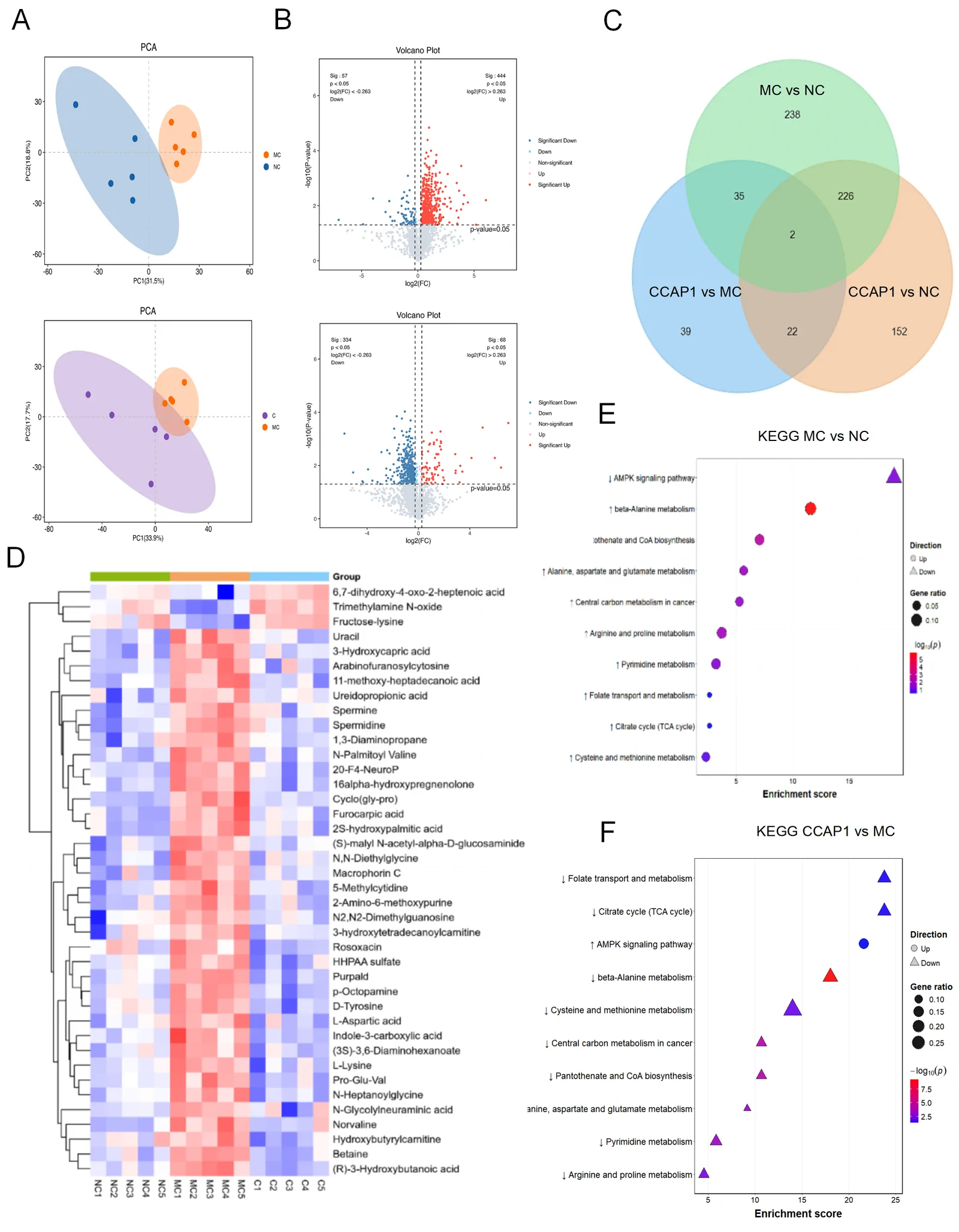
Untargeted skin metabolomics reveals CCAP1-induced metabolic remodeling in UVB-exposed mouse skin. (**A**) Principal component analysis (PCA) score plots of the skin metabolome (*n* = 5). (**B**) Volcano plots of differential metabolites (upper, MC vs. NC; lower, CCAP1 vs. MC); differential metabolites were screened by *p* < 0.05 and |log_2_FC| > 0.263, with thresholds indicated in each panel (*n* = 5). (**C**) Venn diagram showing the numbers of differential metabolites among the pairwise comparisons (MC vs. NC, CCAP1 vs. MC, CCAP1 vs. NC) (*n* = 5). (**D**) Heatmap of the top-ranked differential metabolites across individual samples (*n* = 5 per group). (**E**,**F**) Bubble diagrams of KEGG pathway enrichment for MC vs. NC (**E**) and CCAP1 vs. MC (**F**); dot size indicates the number of enriched metabolites and color indicates enrichment significance (*n* = 5). Arrows before pathway names indicate regulatory direction: ↑, upregulated pathway; ↓, downregulated pathway. AMPK signaling, beta-alanine metabolism, and cysteine–methionine metabolism were consistently enriched in both comparisons. NC, normal control; MC, UVB-irradiated model control; FC, fold change.

To characterize the metabolic basis of CCAP1-mediated photoprotection, untargeted metabolomics was performed on skin tissues from the NC, MC, and CCAP1-H groups. Principal component analysis (PCA) revealed clear separation of the metabolic profiles among the three groups, indicating that UVB irradiation profoundly perturbed the skin metabolome and that CCAP1 partially redirected the metabolic trajectory toward the NC state (Figure 3A). Volcano plot analysis showed that, compared with the NC group, 444 metabolites were significantly up-regulated and 57 down-regulated in the MC group; compared with the MC group, 58 metabolites were significantly up-regulated and 334 down-regulated in the CCAP1 group (Figure 3B). Venn analysis further identified 37, 228, and 24 differential metabolites in the pairwise comparisons among the three groups (Figure 3C), and the overall abundance patterns of the key differential metabolites are summarized in the heatmap (Figure 3D).

KEGG enrichment analysis revealed that UVB irradiation significantly perturbed multiple pathways, including the down-regulated AMPK signaling pathway, as well as the up-regulated beta-alanine metabolism, pantothenate and CoA biosynthesis, alanine, aspartate, glutamate metabolism, pyrimidine metabolism, the TCA cycle, and cysteine and methionine metabolism (Figure 3E). In the CCAP1 vs. MC comparison, CCAP1 treatment significantly up-regulated the AMPK signaling pathway while down-regulating the UVB-driven beta-alanine metabolism, cysteine and methionine metabolism, pyrimidine metabolism, the TCA cycle, and arginine and proline metabolism (Figure 3F). As a master sensor of cellular energy and redox status, AMPK was therefore identified as the central hub linking UVB-associated metabolic rewiring, particularly amino acid, polyamine, and pyrimidine metabolism, to skin photoprotection.

### 2.6. Regulation of the AMPK/Nrf2/NF-κB Axis by CCAP1 in Mouse Skin and Metabolomic Correlation Analysis

**Figure 4:**
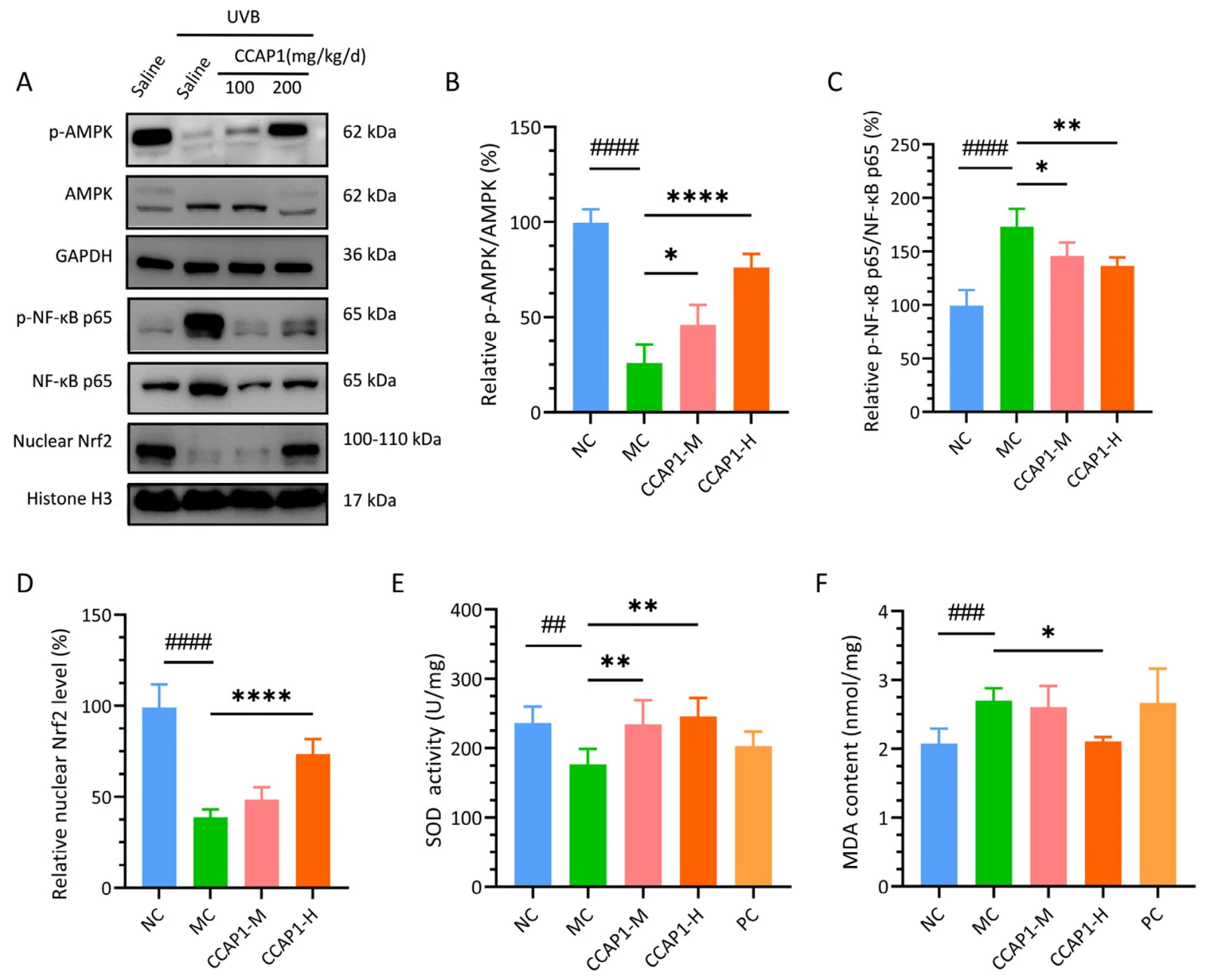
CCAP1 activates AMPK and restores antioxidant defense in UVB-exposed skin. (**A**) Representative Western blot images showing the protein expression levels of p-AMPK, AMPK, p-NF-κB p65, NF-κB p65, Nuclear Nrf2, and their corresponding internal references (GAPDH for cytoplasmic proteins and Histone H3 for nuclear proteins) (*n* = 5). (**B**–**D**) Quantitative analysis of the relative protein levels of p-AMPK/AMPK (**B**), p-NF-κB p65/NF-κB p65 (**C**), and Nuclear Nrf2 (**D**) based on Western blot bands (*n* = 5). (**E**,**F**) The contents of SOD (**E**) and MDA (**F**) in skin tissues (*n* = 10). ^##^ *p* < 0.01, ^###^ *p* < 0.001, ^####^ *p* < 0.0001 vs. NC group; * *p* < 0.05, ** *p* < 0.01, **** *p* < 0.0001 vs. MC group. NC: normal control; MC: model control (UVB-exposed); CCAP1-M: medium-dose CCAP1; CCAP1-H: high-dose CCAP1; PC: positive control.

To verify the metabolomics-nominated AMPK pathway at the protein level, Western blotting was performed to examine key proteins of the AMPK/Nrf2/NF-κB axis in UVB-damaged skin (Figure 4A–D). UVB irradiation significantly reduced the p-AMPK/AMPK ratio and nuclear Nrf2 level while elevating the p-NF-κB p65/NF-κB p65 ratio (*p* < 0.0001). Oral administration of CCAP1 at the medium dose (100 mg/kg/d) significantly restored the p-AMPK/AMPK ratio (*p* < 0.05) and nuclear Nrf2 level (*p* < 0.01), and markedly suppressed NF-κB p65 phosphorylation (*p* < 0.0001); the high dose (200 mg/kg/d) further restored p-AMPK/AMPK and nuclear Nrf2 (*p* < 0.0001) while significantly inhibiting NF-κB p65 phosphorylation (*p* < 0.0001). Consistently, high-dose CCAP1 significantly elevated skin SOD activity (*p* < 0.01) and reduced MDA content (*p* < 0.05) in UVB-damaged skin (Figure 4E,F), corroborating the activation of the Nrf2-dependent antioxidant program.

**Figure 5:**
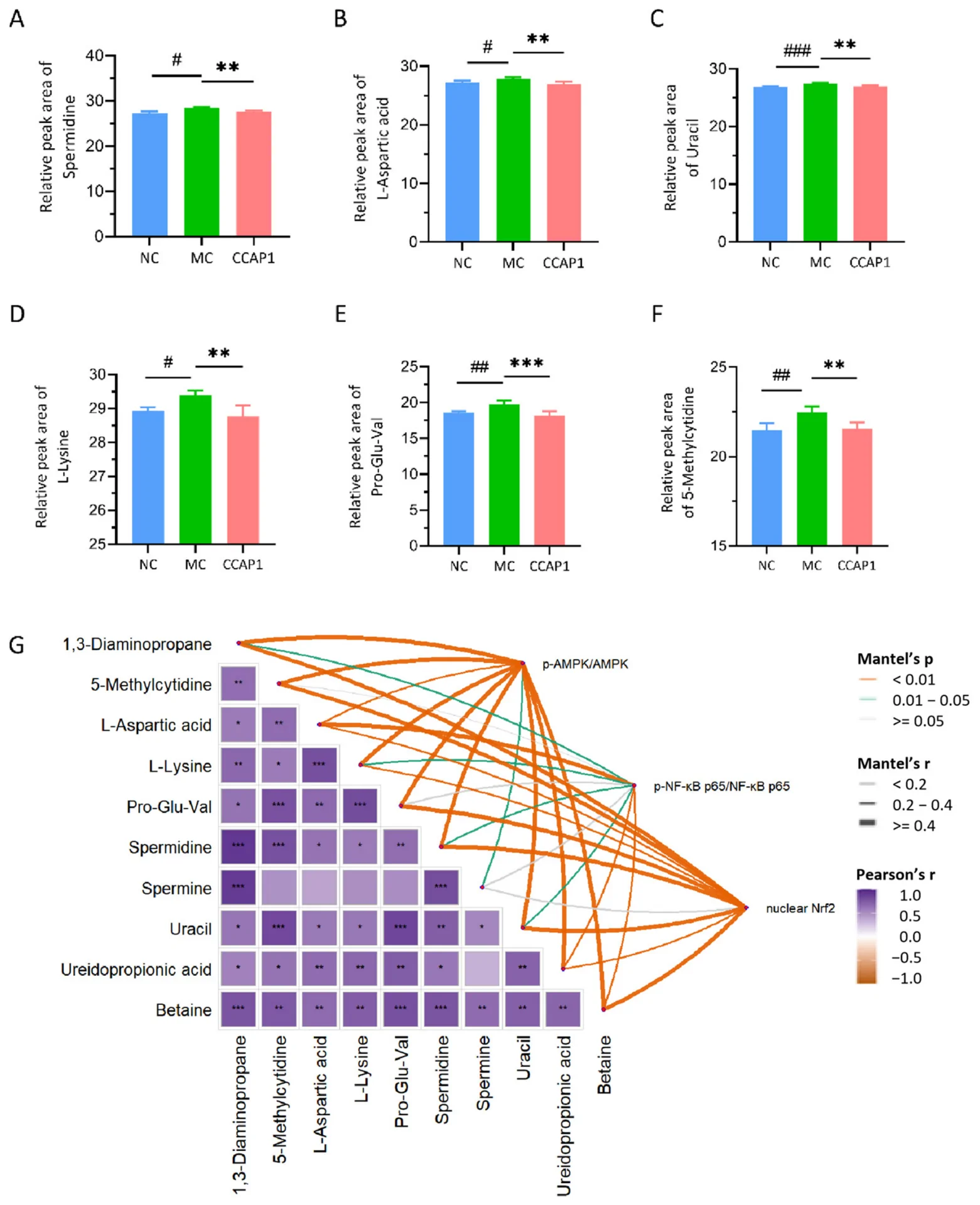
Targeted validation of representative differential metabolites and correlation analysis between metabolites and AMPK/Nrf2/NF-κB signaling proteins. (**A**–**F**) Relative peak areas of six representative differential metabolites in skin tissues from the NC, MC, and CCAP1 groups (*n* = 5): spermidine (**A**), L-aspartic acid (**B**), uracil (**C**), L-lysine (**D**), Pro-Glu-Val (**E**), and 5-methylcytidine (**F**). Data are presented as mean ± SD. ^#^ *p* < 0.05, ^##^ *p* < 0.01, ^###^ *p* < 0.001 vs. NC group; * *p* < 0.05, ** *p* < 0.01, *** *p* < 0.001 vs. MC group. (**G**) Correlation analysis between differential metabolites and key protein markers (*n* = 5). Mantel test was applied to evaluate the overall association between metabolomic profiles and the signaling protein matrix. Pearson correlation analysis was used to calculate pairwise correlation coefficients. Associations with *p* < 0.05 were considered statistically significant.

Ten photoaging-related differential metabolites were subjected to Pearson correlation analysis. Six representative metabolites were visualized in Figure 5A–F according to pathway enrichment and biological relevance, while the other four metabolites were only used in correlation analysis. UVB irradiation markedly increased the abundances of these metabolites in model mice, whereas CCAP1 treatment effectively reversed such metabolic disturbance and restored metabolite levels close to those of the normal control group. The altered metabolites fall into three major metabolic categories: polyamine metabolism, amino acid and peptide metabolism, and β-alanine and pyrimidine metabolism, represented by spermidine, L-aspartic acid, uracil, L-lysine, Pro-Glu-Val, and 5-methylcytidine. Mantel test and Pearson correlation analysis uncovered the association pattern between metabolites and signaling pathways within UVB-disordered metabolic routes, and these associations were statistically significant (Figure 5G). The enrichment of metabolites related to polyamine, β-alanine and pyrimidine was reduced when AMPK and Nrf2 signaling were activated, while these metabolites accumulated substantially upon elevated NF-κB p65 phosphorylation. Specifically, the levels of these metabolites were negatively correlated with the p-AMPK/AMPK ratio and nuclear Nrf2 abundance, and positively correlated with p-NF-κB p65 levels. Collectively, these results demonstrate that CCAP1-mediated metabolic restoration is tightly coupled with AMPK activation, Nrf2 nuclear translocation and NF-κB inhibition, supporting the AMPK-centered regulatory mechanism underlying the photoprotective effect of CCAP1.

### 2.7. Compound C Abolishes CCAP1-Mediated AMPK Activation and Cytoprotection in UVB-Damaged HaCaT Cells

**Figure 6:**
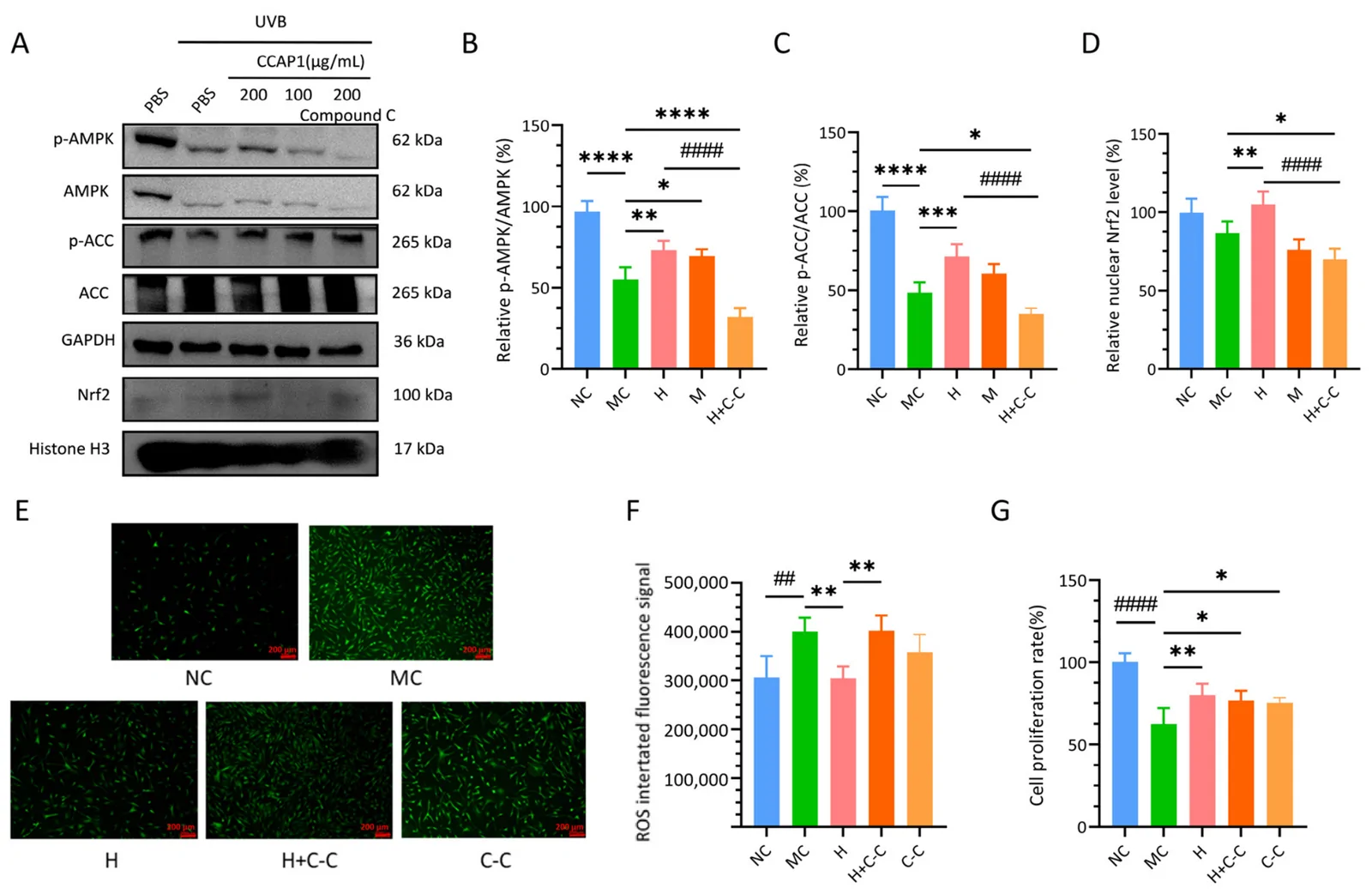
Compound C abrogates CCAP1-mediated AMPK activation and cytoprotection in UVB-irradiated HaCaT cells. (**A**) Representative Western blot images of p-AMPK, AMPK, p-ACC, ACC, and Nuclear Nrf2 in HaCaT cells under the indicated treatments (*n* = 5). (**B**,**C**) Quantitative analysis of p-AMPK/AMPK (**B**), p-ACC/ACC (**C**), and Nuclear Nrf2 (**D**) (*n* = 5). HaCaT cells were pretreated with CCAP1 (100 μg/mL, M; 200 μg/mL, H) for 24 h with or without the AMPK inhibitor Compound C (10 μM, H+C-C), followed by UVB irradiation (30 mJ/cm^2^). (**E**) Representative DCFH-DA fluorescence images showing intracellular ROS levels (*n* = 5). (**F**) Quantitative analysis of ROS fluorescence intensity (*n* = 5). (**G**) Cell viability assessed by CCK-8 assay (*n* = 5). Data are presented as mean ± SD. * *p* < 0.05, ** *p* < 0.01, *** *p* < 0.001, **** *p* < 0.0001 vs. MC; ^##^ *p* < 0.01, ^####^ *p* < 0.0001 vs. CCAP1-H group. NC, negative control; MC, UVB-irradiated model control; H, 200 μg/mL CCAP1; M, 100 μg/mL CCAP1; H+C-C, 200 μg/mL CCAP1 + Compound C; C-C, Compound C.

To determine whether the protective effects of CCAP1 depend on AMPK activation, loss-of-function experiments were performed in UVB-irradiated HaCaT keratinocytes using the AMPK inhibitor Compound C. Compared with the NC group, UVB exposure markedly reduced p-AMPK, Nrf2 nuclear and its direct downstream substrate ACC (*p* < 0.0001). Relative to the MC group, 200 μg/mL CCAP1 significantly restored p-AMPK (*p* < 0.01) and p-ACC (*p* < 0.001) levels in UVB-damaged cells, with a significantly greater effect than 100 μg/mL (*p* < 0.05). Co-administration of 200 μg/mL CCAP1 with Compound C (10 μM, H+C-C group) markedly decreased both p-AMPK and p-ACC relative to the H group (*p* < 0.0001), and their levels fell below those of the MC group (Figure 6). Given that the downstream NF-κB readouts were validated in UVB-exposed mouse skin, a loss-of-function experiment was designed to test the causal necessity of AMPK itself; whether Compound C also abolishes CCAP1-induced NF-κB suppression in HaCaT cells warrants further investigation. Collectively, these findings support AMPK as a key upstream mediator of CCAP1 photoprotection and provide a mechanistic link between AMPK activation and the in vivo restoration of redox homeostasis and preservation of dermal collagen integrity.

Consistent with the molecular findings, CCAP1 pretreatment significantly improved cell viability and suppressed intracellular ROS accumulation in UVB-damaged HaCaT cells (Figure 6D–F). However, these antioxidant and cytoprotective effects were largely abrogated by Compound C co-treatment, as evidenced by a significant increase in DCFH-DA fluorescence and a concurrent reduction in cell viability. Compound C treatment alone did not significantly alter ROS levels or cell viability compared with the UVB model group. Collectively, these results demonstrate that CCAP1 attenuates UVB-induced oxidative damage in HaCaT keratinocytes through an AMPK-dependent mechanism.

## 3. Discussion

In this study, we prepared CCAP1, a collagen-derived peptide fraction comprising 94.69% collagen-related components, from *Colla Corii Asini* (CCA) and demonstrated its multifaceted anti-photoaging activity in a UVB-challenged mouse model and in HaCaT keratinocytes. Our findings indicate that CCAP1 acts through an AMPK-centered regulatory mechanism that promotes Nrf2 activation and suppresses NF-κB p65 phosphorylation, thereby alleviating oxidative stress, preserving dermal collagen, and ameliorating UVB-induced skin photoaging. These results position CCAP1 as a promising composition-defined natural ingredient with translational potential for oral skin photoprotection.

Untargeted skin metabolomics revealed that CCAP1 triggers metabolic reprogramming consistent with AMPK-mediated regulatory patterns, providing a system-level view underlying its photoprotective mechanism. The proposed signaling paradigm positions AMPK as the central metabolic sensor that coordinates cutaneous redox homeostasis with NF-κB signaling [22,23], and is consistent with reports that collagen-derived peptides modulate energy-sensing pathways in skin cell systems [24,25,26]. Western blot analyses of mouse skin tissues demonstrated that AMPK activation coincided with enhanced nuclear Nrf2 accumulation and suppressed NF-κB signaling under UVB challenge. At the functional level, CCAP1 restored cell viability and reduced intracellular ROS in UVB-damaged HaCaT keratinocytes; both effects were abolished by pharmacological AMPK inhibition with Compound C, directly demonstrating that the antioxidant and cytoprotective functions of CCAP1 are AMPK-dependent. Notably, spermidine—although generally regarded as a geroprotective metabolite—accumulates in UVB-challenged skin, consistent with UVB-induced ornithine decarboxylase activation and epidermal hyperproliferation [27]. CCAP1 restored polyamine homeostasis concomitant with the suppression of epidermal hyperplasia and oxidative stress. By integrating unbiased metabolomic screening, in vivo molecular validation, and in vitro pharmacological inhibition experiments, this study provides a causal evidence chain linking the defined oligopeptide composition of CCAP1 to its anti-photoaging efficacy via the AMPK-Nrf2/NF-κB axis.

Consistent with previous studies, collagen-derived bioactive peptides modulate cutaneous redox balance and downstream signaling to confer anti-photoaging effects [13,28,29]. However, two major gaps remain. First, downstream pathways such as TGF-β/Smad and MAPK/NF-κB dominate the literature, whereas the upstream regulatory hub governing photoprotection remains poorly defined [12,30]. Second, previous studies on *Colla Corii Asini*-derived peptides often rely on poorly characterized crude mixtures or focus on non-collagen monomeric sequences, with limited structure–activity understanding and upstream mechanistic evidence [20]. In contrast, CCAP1 reprograms cellular metabolism with AMPK at the center, coordinating Nrf2 antioxidant responses with repression of NF-κB signaling, yielding an AMPK-dominated regulatory signature distinct from previously reported collagen peptides. Methodologically, the integration of untargeted metabolomics with pharmacological inhibition in HaCaT cells moves beyond single-pathway validation and establishes a causal link between oral CCAP1 administration and AMPK-mediated photoprotection. Dorsomorphin (Compound C) is a non-selective kinase inhibitor with off-target effects that may complicate data interpretation. Our pharmacological results support a contributory role of AMPK, though genetic perturbation is needed for definitive causal proof. We focused on the AMPK–Nrf2 axis here; further NF-κB p65 assays are required to validate their AMPK-dependent crosstalk. Moreover, unlike the topical collagen-peptide approach of Wu et al. [31], our findings support the oral efficacy and translational potential of CCAP1, providing a repeatable framework for natural peptide-based skin photoprotection.

CCAP1 exhibits direct free-radical scavenging activity in vitro (79.28% superoxide scavenging at 200 μg/mL); however, its in vivo photoprotective effect is achieved primarily through AMPK-dependent metabolic and signaling reprogramming rather than stoichiometric radical quenching alone. This dual-mode profile distinguishes CCAP1 from previously reported collagen peptides and provides a coherent mechanistic link between metabolic remodeling and photoprotection. From a translational perspective, the mouse dose of 100–200 mg/kg/day corresponds to a human-equivalent dose of roughly 8.1–16.2 mg/kg/day, based on body surface area conversion factors [16,32]. In parallel, the everted gut sac model demonstrates favorable jejunal permeability, and 28 days of administration produced no detectable changes in body weight or organ coefficients, supporting the oral safety and practicality of CCAP1 for UV-exposed populations. Definitive efficacy and safety in humans, however, require controlled dietary intervention studies.

Several limitations should be acknowledged. First, CCAP1 is a complex oligopeptide mixture, which precluded pharmacokinetic quantification of individual peptides in plasma [33]; nevertheless, the restoration of dermal collagen and the global metabolic reprogramming observed in skin tissue provide indirect evidence that orally administered CCAP1, or its bioavailable digestion products, reached the systemic circulation. Second, peptide quantification in the everted gut sac assay was based on BCA; although this approach is widely used for peptide mixture permeability assessment, it does not track individual peptide species, and future studies employing isotope-labeled or signature-peptide tracing would provide more precise absorption kinetics [34]. Third, while Compound C-mediated AMPK inhibition provides pharmacological evidence for a causal role of AMPK, Compound C is not fully selective for AMPK, and genetic approaches (e.g., siRNA-mediated AMPK knockdown or CRISPR-based knockout) were not performed; such experiments, together with in vivo AMPK activation/knockout models, will be important for definitive mechanistic confirmation. Fourth, the metabolomic analysis was confined to skin tissue; parallel serum metabolomics would enable tracing of the systemic metabolic fate of CCAP1. Finally, the 28-day monitoring of body weight and organ coefficients constitutes a preliminary safety assessment rather than a formal toxicological evaluation, and the murine photoaging model cannot fully recapitulate human skin physiology; controlled human dietary intervention studies are therefore required.

## 4. Materials and Methods

### 4.1. Materials

*Colla Corii Asini* (CCA) was obtained from Dong-E E-Jiao Co., Ltd. (Shandong, China). Flavourzyme, bromelain, papain, and neutral protease were purchased from Novozymes (Beijing, China). HaCaT keratinocytes were acquired from Procell Life Science & Technology Co., Ltd. (Wuhan, China). Compound C (dorsomorphin) was supplied by MedChemExpress (Monmouth Junction, NJ, USA). DMEM medium, fetal bovine serum (FBS), penicillin–streptomycin, and phosphate-buffered saline (PBS) were purchased from Gibco (Grand Island, NY, USA). RIPA lysis buffer, the PAGE Color Gel Rapid Preparation Kit, and the ECL kit were from Servicebio (Wuhan, China); the BCA protein assay kit was from Beyotime (Shanghai, China). Assay kits for superoxide radical scavenging activity, GSH, GSH-Px, SOD, MDA, hydroxyproline (Hyp), collagen types I/III/IV, and MMP1/MMP2/MMP9 were supplied by Nanjing Jiancheng Bioengineering Institute (Nanjing, China). LC-MS grade acetonitrile, methanol, and formic acid were from Thermo Fisher Scientific (Waltham, MA, USA). All other reagents were of analytical grade.

### 4.2. Preparation and Basic Characterization of CCAP1

CCA powder was uniformly dispersed in distilled water at a solid–liquid ratio of 1:20 (*w*/*v*). The mixture was hydrolyzed for 4 h using a combined enzymatic system consisting of 4500 U/g bromelain and 1500 U/g flavourzyme, yielding a total enzyme activity of 6000 U/g substrate at an activity ratio of 3:1. The hydrolysate was inactivated at 100 °C for 10 min and centrifuged at 8000× *g* for 15 min, and the supernatant was collected. After purification via 3 kDa ultrafiltration and 200 Da desalting nanofiltration, the refined collagen peptide fraction was obtained and designated CCAP1. The total protein content of CCAP1 was determined by the Kjeldahl method, while hydroxyproline (Hyp) content was quantified following the corresponding national standard. The molecular weight distribution of CCAP1 was determined by gel permeation chromatography (GPC) using a TSKgel G2000 SWXL column (7.8 × 300 mm) with ultrapure water as the mobile phase at a flow rate of 0.5 mL/min and a detection wavelength of 220 nm, calibrated with standard molecular weight markers. The superoxide radical scavenging activity was measured at 550 nm according to the manufacturer’s instructions [35]. All experiments were performed in triplicate.

### 4.3. LC-MS/MS Identification of Characteristic Oligopeptides in CCAP1

CCAP1 was desalted using C18 Spin Tips and analyzed by UHPLC-Q-Orbitrap MS. A 5 μL sample was separated on a C18 capillary column (20 cm × 75 μm i.d., 1.9 μm) at 300 nL/min and 40 °C. Mobile phase A was 0.1% formic acid in water, and mobile phase B was 80% acetonitrile containing 0.1% formic acid. The gradient elution was 5–72% B over 57 min, followed by 3 min of 99% B for column washing. Electrospray voltage was set at 2.0 kV, and MS data were acquired in positive DDA mode. Full MS scans (*m*/*z* 200-1550) were collected at 120,000 resolution (AGC 300%, max injection 20 ms), and HCD MS/MS spectra were acquired at 15,000 resolution (AGC 200%, max injection 22 ms, NCE 32%, dynamic exclusion 35 s). Raw spectra were searched against the *Equus asinus* UniProt database (2025, 21,403 entries) using PEAKS Studio 13 with unspecific digestion. Precursor and fragment mass tolerances were 10 ppm and 0.02 Da, respectively. Variable modifications included methionine oxidation and asparagine/glutamine deamidation. Peptides with PSM −10lg*P* ≥ 20 and at least one unique peptide were retained. Dominant oligopeptides were characterized by retention time, peptide length, and *m*/*z*.

### 4.4. Everted Gut Sac Assay for Intestinal Absorption of CCAP1

The CCAP1 solution was sequentially digested with simulated gastric fluid (pH 2.0, pepsin, 37 °C, 2 h) and simulated intestinal fluid (pH 7.0, trypsin, 37 °C, 2 h) [36]. Female SD rats (6–8 weeks old) were fasted for 12 h and sacrificed. Segments of duodenum, jejunum and ileum were dissected, everted, filled with blank Krebs–Ringer buffer (KRB), and immersed in KRB containing the digested CCAP1. The system was incubated at 37 °C with constant shaking for 90 min; six independent biological replicates (*n* = 6) were conducted. The apparent permeability coefficient (*P_app_*) was calculated to evaluate the intestinal absorption of CCAP1 across the duodenum, jejunum and ileum. All animal procedures were approved by the Institutional Animal Care and Use Committee of Ocean University of China (approval No. E-MBAD-2025-3-20).

### 4.5. Establishment of UVB-Induced Skin Photoaging Model and Oral Intervention

Specific-pathogen-free (SPF) female BALB/c mice (6–8 weeks old, 18–22 g) were housed under standardized laboratory conditions (12 h light/dark cycle, 22 ± 2 °C, 55 ± 5% relative humidity) with free access to standard diet and drinking water. After one week of adaptive feeding, mice were randomly divided into five groups (*n* = 10): normal control (NC, saline treatment without UVB irradiation), model control (MC, saline gavage + UVB irradiation), medium-dose CCAP1 (CCAP1-M, 100 mg/kg/day CCAP1+UVB), high-dose CCAP1 (CCAP1-H, 200 mg/kg/day CCAP1+UVB), and positive control (PC, 100 mg/kg/day ergothioneine + UVB). One day before UVB exposure, dorsal hair of all mice except the NC group was completely shaved. Mice were irradiated with UVB light (312 nm, 1200 mJ/cm^2^ per session, 45 min) five times per week for six consecutive weeks. After two weeks of continuous UVB pre-stimulation, mice received daily oral gavage administration of corresponding treatments for 28 days, with UVB irradiation maintained throughout the intervention period. Body weight was recorded weekly.

On day 28, dorsal skin hydration was measured on conscious mice (*n* = 10) using a skin moisture detector under controlled temperature and humidity; multiple sites on each dorsal skin were measured and averaged. Mice were then euthanized, and depilated dorsal skin was harvested. Each skin sample was longitudinally bisected: the right half was fixed in 4% paraformaldehyde for histology, and the left half was snap-frozen in liquid nitrogen and stored at −80 °C. From the 10 mice per group, five were randomly selected using a random number generator. The frozen left halves of these five mice were aliquoted for untargeted metabolomics, Western blotting, and biochemical assays (SOD, MDA, Hyp, collagens I/III/IV, MMP1). The frozen left halves of the remaining five mice were used for biochemical assays only. All fixed right halves (*n* = 10) were subjected to histological evaluation (H&E, Masson’s trichrome staining and epidermal thickness quantification). This sample allocation gave skin moisture and histology, *n* = 10; biochemical assays, *n* = 10; and metabolomics and Western blotting, *n* = 5, allowing matched correlation analysis between metabolites and signaling proteins. All animal operations complied with the ethical guidelines approved by the Institutional Animal Care and Use Committee of Ocean University of China (approval No. E-MBAD-2025-3-20).

### 4.6. Macroscopic, Histological, and Phenotypic Evaluation of Skin Tissue

Dorsal skin photographs were captured on day 28 to visually evaluate skin erythema, scaling, dryness, and roughness. Fixed skin specimens were embedded in paraffin and cut into 4 μm continuous sections. Hematoxylin–eosin (H&E) staining was performed to assess epidermal thickness, inflammatory cell infiltration, and hair follicle morphology. Masson’s trichrome staining was applied to observe the distribution and integrity of dermal collagen fibers. Epidermal thickness was quantified using ImageJ software (v1.54f) based on five random non-overlapping fields per section. The hydroxyproline (Hyp) content in skin homogenates was determined using a commercial assay kit to reflect total collagen deposition in dermal tissue.

### 4.7. Untargeted Metabolomic Analysis of Skin Tissue

Frozen skin tissue (50 mg) from the five selected mice per group was homogenized in pre-chilled methanol–water solution, vortexed thoroughly, and centrifuged at 12,000× *g* for 15 min at 4 °C. The supernatant was vacuum-dried and reconstituted for subsequent LC-MS detection. Quality control (QC) samples were prepared by mixing equal volumes of all individual tissue extracts to monitor instrument stability.

Metabolite separation was performed on a Waters ACQUITY UPLC HSS T3 column (100 mm × 2.1 mm, 1.8 μm) at 45 °C, with mobile phases consisting of 0.1% formic acid in water (A) and pure acetonitrile (B) at a flow rate of 0.35 mL/min. Mass spectrometry detection was conducted using a Thermo QE Plus mass spectrometer in both positive and negative ionization modes under DDA acquisition. Raw metabolomic data were processed via Progenesis QI software for peak alignment, normalization, and metabolite annotation [37]. Multivariate statistical analyses, including PCA and OPLS-DA, were performed using SIMCA 14.1. A 200-fold permutation test was conducted to evaluate the robustness of the OPLS-DA model and rule out overfitting. Differential metabolites were screened by a combined criterion: VIP > 1 from OPLS-DA, fold change (FC) ≥ 1.2, and Benjamini–Hochberg false discovery rate (BH-FDR) adjusted *p* < 0.05. Metabolites with RSD < 30% in QC samples were retained for downstream analysis. All identified differential metabolites were annotated against the KEGG database for pathway enrichment analysis [38]. Bubble plots were generated to display the top enriched metabolic pathways.

Mantel test and Pearson correlation analysis were conducted to evaluate the associations between eleven critical differential metabolites and core signaling proteins (p-AMPK/AMPK, p-NF-κB p65/NF-κB p65, and nuclear Nrf2). Because untargeted metabolomics, Western blotting, and antioxidant index detection were all performed on aliquots from the same five selected mice per group, metabolite and protein measurements were matched at the individual animal level for correlation analysis.

### 4.8. Detection of Oxidative Stress Indicators, Collagen Subtypes and Matrix Metalloproteinases

Skin homogenates were prepared from frozen skin tissue in pre-cooled PBS and centrifuged to collect supernatants. The oxidative stress-related indices, including reduced glutathione (GSH) content, glutathione peroxidase (GSH-Px) activity, superoxide dismutase (SOD) activity, and malondialdehyde (MDA) level, were measured using commercial assay kits in strict accordance with the manufacturer’s instructions. Meanwhile, the contents of type I, III, and IV collagen, as well as matrix metalloproteinases (MMP1, MMP2, and MMP9), in skin homogenates were quantified via corresponding ELISA kits. The absorbance values of all samples were detected using a microplate reader, and the corresponding substance concentrations or enzyme activities were calculated based on standard curves. All biochemical assays were performed with *n* = 10 biological replicates per group.

### 4.9. AMPK Pharmacological Inhibition in UVB-Irradiated HaCaT Keratinocytes

HaCaT keratinocytes were cultured in DMEM medium supplemented with 10% FBS and 1% penicillin–streptomycin in a humidified incubator with 5% CO_2_ at 37 °C. Cells were seeded in 6-well plates and divided into six groups: negative control (NC), UVB-induced model control (MC), medium-dose CCAP1 (100 μg/mL), high-dose CCAP1 (200 μg/mL), high-dose CCAP1 combined with Compound C (H+C-C), and Compound C alone (C-C). Cells in CCAP1 pretreatment groups were incubated with the corresponding concentrations of CCAP1 for 24 h. For UVB stimulation, cells were rinsed with PBS, covered with a thin layer of PBS, and irradiated with 30 mJ/cm^2^ UVB (312 nm). For the H+C-C and C-C groups, 10 μM Compound C was added 1 h before UVB irradiation and maintained during subsequent incubation. After UVB exposure, cells were cultured in serum-free DMEM with the corresponding treatments for 24 h.

Cell viability was determined using the CCK-8 assay. CCK-8 working solution was added to each well and incubated at 37 °C. The absorbance was measured at 450 nm using a microplate reader. Intracellular ROS levels were detected by the DCFH-DA fluorescent probe. After treatment, cells were incubated with DCFH-DA working solution in the dark. The fluorescence intensity was captured by a fluorescence microscope, and quantitative fluorescence values were determined using a microplate reader. Subsequently, cells were harvested for Western blot analysis to detect the expression of p-AMPK/AMPK, nuclear Nrf2 and p-ACC/ACC.

### 4.10. Western Blot Analysis

Skin tissues and cultured HaCaT cells were lysed using RIPA lysis buffer supplemented with protease and phosphatase inhibitors. Equal amounts of total protein (30 μg) were separated by SDS-PAGE and electrotransferred onto PVDF membranes. After blocking with 5% non-fat milk for 1 h, membranes were incubated with primary antibodies against p-AMPKα (Thr172), AMPKα, p-ACC (Ser79), ACC, p-NF-κB p65 (Ser536), NF-κB p65, Nrf2, GAPDH, and Histone H3 overnight at 4 °C. Subsequently, membranes were incubated with HRP-conjugated secondary antibodies at room temperature. Protein bands were visualized using an ECL chemiluminescence system and quantified via ImageJ software. GAPDH and Histone H3 were used as internal references for cytoplasmic and nuclear proteins, respectively.

### 4.11. Statistical Analysis

All experimental data are expressed as mean ± standard deviation (SD). The sample size for each assay is specified in Section 4.5 and in the corresponding figure legends. GraphPad Prism 9.0 was used for statistical comparison among multiple groups. One-way analysis of variance (ANOVA) followed by Tukey’s multiple comparison test was adopted for intergroup comparisons. Pearson correlation analysis and the Mantel test were implemented in R software. A *p*-value or adjusted *p*-value < 0.05 was defined as statistically significant.

## 5. Conclusions

In summary, we prepared and characterized CCAP1, a well-defined type I collagen-rich oligopeptide fraction from *Colla Corii Asini*. CCAP1 exhibited favorable intestinal permeability and satisfactory 28-day oral tolerability in UVB-exposed mice. Oral CCAP1 effectively mitigated UVB-triggered skin photoaging by alleviating epidermal hyperplasia, restoring skin hydration and dermal collagen deposition, and inhibiting MMP1-mediated collagen breakdown. Mechanistically, integrated untargeted metabolomics, Western blotting and pharmacological inhibition assays demonstrated that CCAP1 exerts photoprotective effects through an AMPK-dependent regulatory cascade, which activates Nrf2 antioxidant responses and suppresses NF-κB signaling. This study identifies CCAP1 as a promising orally bioactive natural ingredient for skin photoprotection.

## Figures and Tables

**Table 1:** Protein composition profile of CCAP1 based on peptidomic quantification.

Protein Name	Accession	Relative Protein Abundance (%)
COL1A1	A0A9L0IR78	49.62
COL1A2	A0A8C4MSS5	25.49
COL3A1	A0A9L0KDG1	7.38
COL5A2	A0A8C4MTB6	1.94
Other collagen-related proteins	--	10.26
Non-collagen proteins	--	5.31
Total		100

**Table 2:** The top 10 most abundant peptides in CCAP1 identified by UHPLC-Q-Orbitrap MS.

Peptide Sequence	Theoretical Mass	Length	*m*/*z*	RT	Relative Peak Area%
GPLGPP(+15.99)GP	706.36	8	707.3716	15.9144	3.63%
GPIGPP(+15.99)GP	706.36	8	707.3716	15.9144	3.63%
P(+15.99)GPAGP	510.2389	6	511.2511	57.4545	2.53%
QPP(+15.99)GP	510.2389	5	511.251	3.1903	2.40%
PGP(+15.99)GAP	510.2389	6	511.2509	4.9654	2.40%
QPAGP	468.2332	5	469.2406	55.5571	2.30%
GEAGRP(+15.99)GEAGL P(+15.99)GAKG	1454.702	16	728.3629	7.7268	1.74%
PGVGP	425.2274	5	426.2357	38.2589	1.60%
GPAGPP(+15.99)GFPG	868.403	10	869.4156	24.7915	1.48%
GERGPP(+15.99)GP	781.3669	8	391.6931	4.6958	1.38%

## Data Availability

The original contributions presented in this study are included in the article/supplementary material. Further inquiries can be directed to the corresponding authors.

## Supplementary Materials

The following supporting information can be downloaded at: https://www.mdpi.com/article/10.3390/molecules31213964/s1. Figure S1: MS/MS spectra of the ten most abundant oligopeptides identified from CCAP1. Figure S2: Activity-guided preparation and characterization of CCAP1. Figure S3: Organ indices of mice after the 28-day oral intervention. Figure S4: Effects of CCAP1 on matrix metalloproteinase (MMP) levels in UVB-exposed mouse skin. Figure S5: Effects of CCAP1 on additional antioxidant markers in UVB-exposed mouse skin. Table S1: Complete list of proteins identified in CCAP1 by UHPLC-Q-Orbitrap MS/MS peptidomic analysis.

## Abbreviations

The following abbreviations are used in this manuscript:

UVB	ultraviolet B
AMPK	5′-AMP-activated protein kinase
NF-κB	nuclear factor kappa-B
Nrf2	nuclear factor erythroid 2-related factor 2
SOD	superoxide dismutase
MDA	malondialdehyde
GPC	gel permeation chromatography
KEGG	Kyoto Encyclopedia of Genes and Genomes
MMP	matrix metalloproteinase
*m*/*z*	mass-to-charge ratio
