# Supplementary material for: A Collagen-Derived Peptide Fraction CCAP1 from Colla Corii Asini Attenuates UVB-Induced Skin Photoaging via an AMPK-Dependent Nrf2/NF-κB Axis
Source: Molecules. 2026 Nov 3;31(21):3964. doi: 10.3390/molecules31213964 (PMC13765522; doi:10.3390/molecules31213964)
Supplement: Supplementary file 1 [file molecules-31-03964-s001.zip › molecules-4617372-supplementary.pdf]

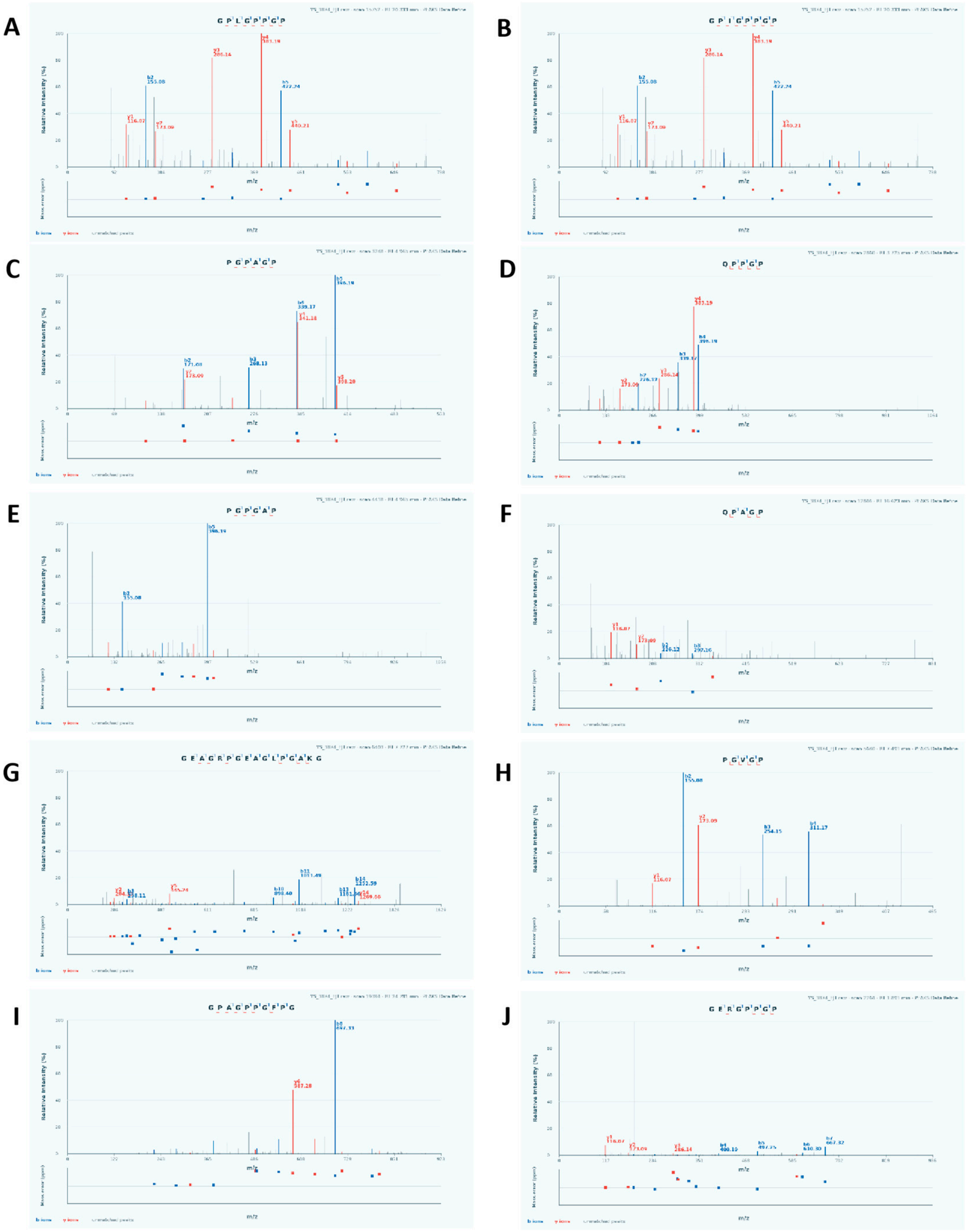

Figure S1: MS/MS spectra of the ten most abundant oligopeptides identified from CCAP1. **(A–J)** Representative tandem mass spectra corresponding to the top 10 peptides listed in Table 2, supporting sequence identification.

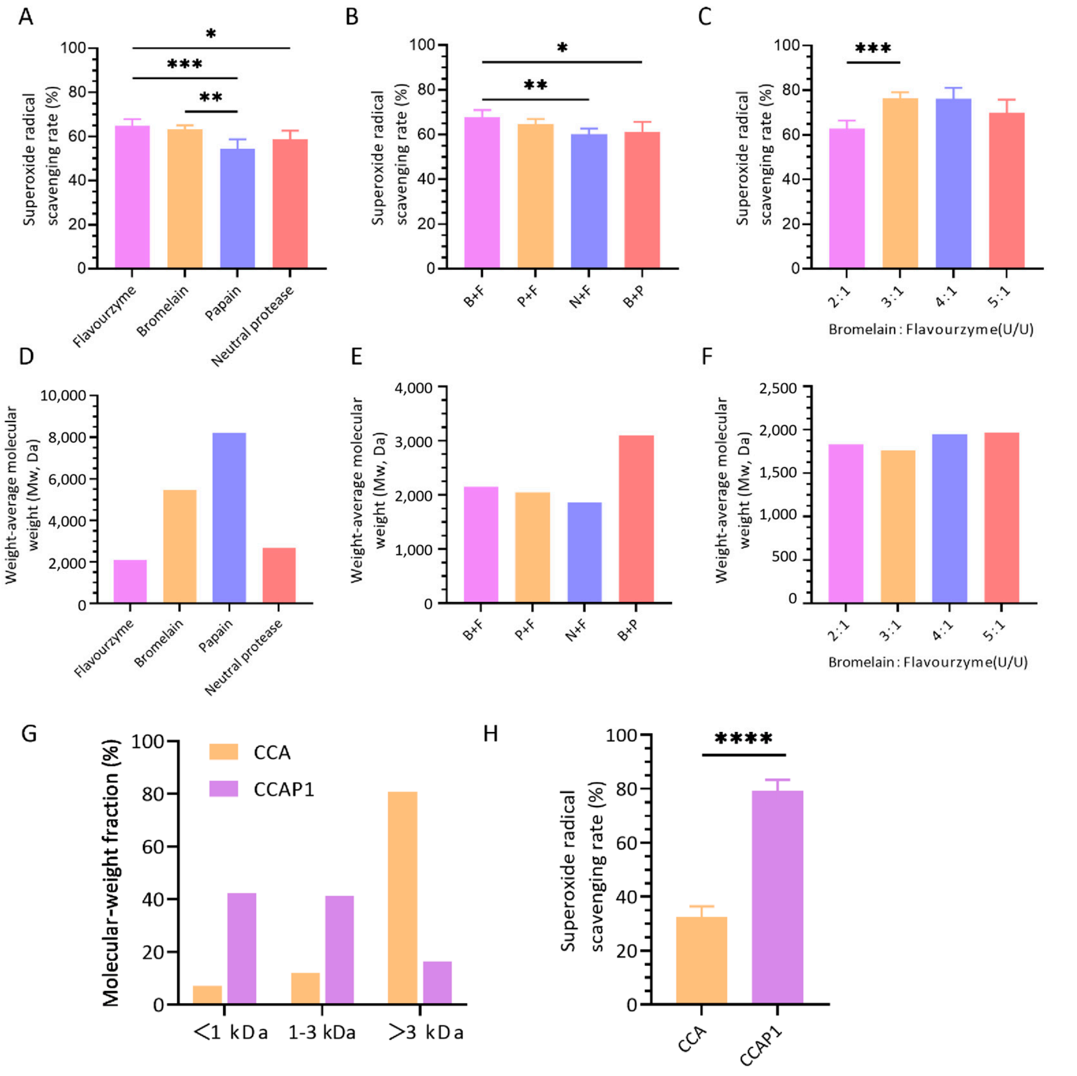

Figure S2: Activity-guided preparation and characterization of CCAP1. **(A–C)** Superoxide radical scavenging rate of CCA hydrolysates prepared with individual proteases **(A)**, binary enzyme combinations **(B)**, and different Bromelain:Flavourzyme ratios **(C)**. **(D–F)** Weight-average molecular weight (*Mw*) of the corresponding hydrolysates. **(G)** Molecular-weight distribution of CCA and CCAP1. **(H)** Superoxide radical scavenging rate of CCA and CCAP1 at 200 μg/mL. Data are mean ± SD ($n = 6$). *$p < 0.05$, **$p < 0.01$, ***$p < 0.001$, ****$p < 0.0001$. CCA, *Colla Corii Asini*; CCAP1, CCA-derived collagen peptide fraction 1; Mw, weight-average molecular weight.

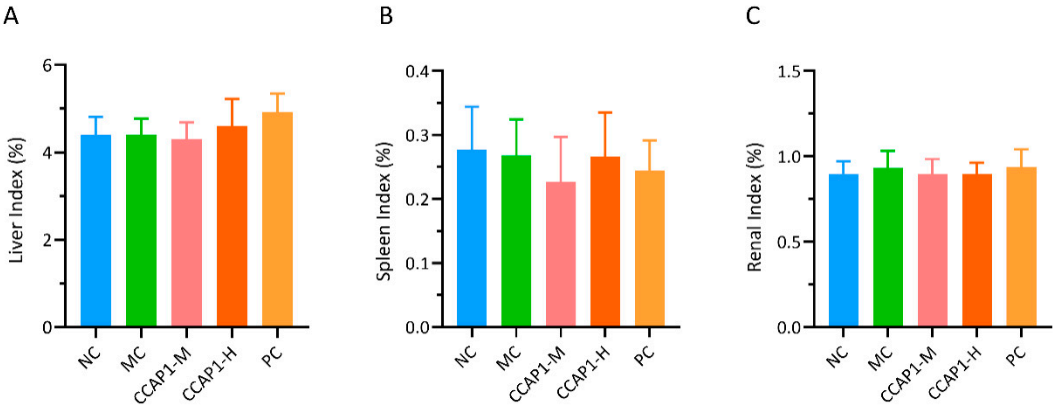

Figure S3: Organ indices of mice after the 28-day oral intervention.
(A) Liver index. **(B)** Spleen index. **(C)** Renal index. Data are presented as mean ± SD (n = 10). No statistically significant differences were observed among the five groups (one-way ANOVA). NC, normal control; MC, UVB-irradiated model control; CCAP1-M, 100 mg/kg/day; CCAP1-H, 200 mg/kg/day; PC, ergothioneine positive control (100 mg/kg/day).

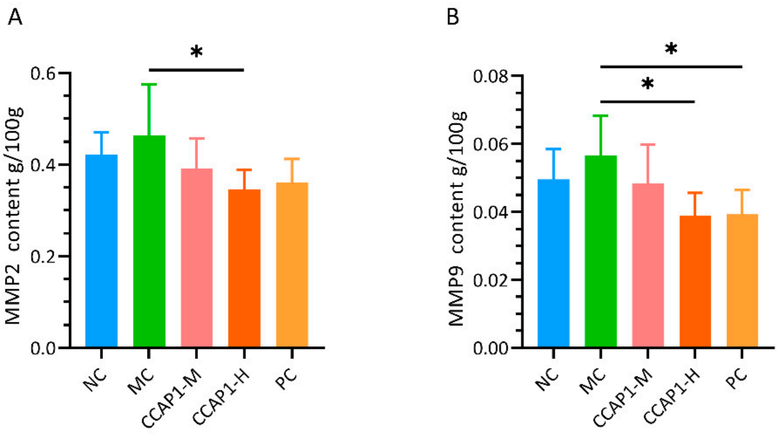

Figure S4: Effects of CCAP1 on matrix metalloproteinase (MMP) levels in UVB-exposed mouse skin.
(A) MMP2 content (g/100 g). **(B)** MMP9 content (g/100 g). Data are presented as mean ± SD (n = 10). *$p < 0.05$ vs. MC; $^{\#}p < 0.05$ vs. NC (as indicated). NC, normal control; MC, UVB-irradiated model control; CCAP1-M, 100 mg/kg/day; CCAP1-H, 200 mg/kg/day; PC, ergothioneine positive control.

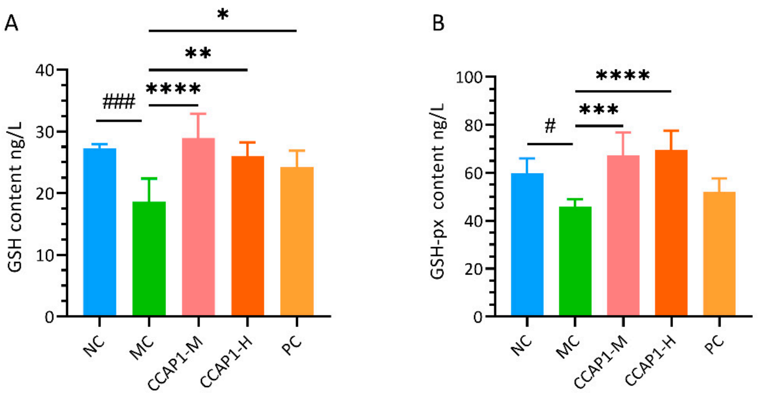

Figure S5: Effects of CCAP1 on additional antioxidant markers in UVB-exposed mouse skin. **(A)** GSH content (ng/L). **(B)** GSH-Px content (ng/L). Data are presented as mean ± SD (n = 10). #$p < 0.05$, ###$p < 0.001$ vs. NC; *$p < 0.05$, **$p < 0.01$, ***$p < 0.001$, ****$p < 0.0001$ vs. MC. NC, normal control; MC, UVB-irradiated model control; CCAP1-M, 100 mg/kg/day; CCAP1-H, 200 mg/kg/day; PC, ergothioneine positive control (100 mg/kg/day).

Table S1: Complete list of proteins identified in CCAP1 by UHPLC-Q-Orbitrap MS/MS peptidomic analysis.

	**Accession**	**Protein name**	**Area**	**Relative protein abundance (%)**
1	A0A9L0IR78	COL1A1	11700000000	49.62
2	A0A8C4MSS5	COL1A2	6010000000	25.49
3	A0A9L0KDG1	COL3A1	1740000000	7.38
4	A0A8C4MTB6	COL5A2	457000000	1.94
5	A0A9L0JVL8	COL5A1	349000000	1.48
6	A0A9L0I5I1	COL11A1	238000000	1.01
7	A0A8C4MZR8	COL2A1	217000000	0.92
8	A0A8C4PM28	COL5A3	200000000	0.85
9	A0A8C4KYX6	COL21A1	169000000	0.72
10	A0A8C4M6Q5	COL17A1	151000000	0.64
11	A0A9L0IBA9	COL18A1	142000000	0.60
12	A0A9L0IIM3	COL4A4	126000000	0.53
13	A0A9L0K4Y6	COL11A2	83100000	0.35
14	A0A9L0IEM0	COL4A5	81800000	0.35
15	A0A9L0JHX3	COLEC12	79500000	0.34
16	A0A9L0K5D2	COL13A1	73600000	0.31
17	A0A8C4N104	COL28A1	63300000	0.27
18	A0A8C4N5R3	COL4A1	60500000	0.26
19	A0A8C4MNC4	COL4A6	53100000	0.23
20	A0A8C4N5G1	COL4A2	51500000	0.22
21	A0A9L0JN67	COL19A1	41700000	0.18

22	A0A9L0IE72	COL4A3	40300000	0.17
23	A0A8C4PHI2	COL12A1	38300000	0.16
24	A0A8C4PKA1	COL10A1	29000000	0.12
25	A0A9L0JUQ9	COL24A1	24700000	0.10
26	A0A9L0J8K2	COL22A1	23700000	0.10
27	A0A9L0JZY3	COL9A3	22300000	0.09
28	A0A9L0IFB2	COL9A1	18200000	0.08
29	A0A9L0ICQ0	COL26A1	15000000	0.06
30	A0A8C4MZZ2	COL14A1	14500000	0.06
31	A0A9L0I779	COL8A2	9880000	0.04
32	A0A8C4LRC9	COL8A1	9600000	0.04
33	A0A9L0J7I9	COL6A1	7230000	0.03
34	A0A9L0JMI6	COL25A1	7800000	0.03
35	A0A8C4LWS0	COL23A1	6820000	0.03
36	A0A8C4KZ16	COL9A2	5330000	0.02
37	A0A9L0KCQ4	COL27A1	5090000	0.02
38	A0A8C4LG60	COL6A3	4360000	0.02
39	A0A8C4N9Q0	COL20A1	4230000	0.02
40	A0A9L0JH12	COL16A1	3060000	0.01
41	A0A8C4PJD0	COL6A2	299000	0.00
42	A0A9L0JP18	FCN3	360000000	1.53
43	A0A9L0KEL3	PERCC1	90700000	0.38
44	A0A9L0J2S1	RHOBTB2	69600000	0.30
45	A0A8C4MVR5	ARID3A	63400000	0.27
46	A0A8C4L0S2	SF3B4	59500000	0.25
47	A0A9L0JRK4	LOC106830661	52500000	0.22
48	A0A8C4LTH6	PEF1	29600000	0.13
49	A0A9L0IHG6	THAP11	23600000	0.10
50	A0A8C4L525	SIPA1	23600000	0.10
51	A0A9L0I6B4		21800000	0.09
52	A0A8C4LS65	INTS5	17800000	0.08
53	A0A9L0JQS5	EMILIN1	16700000	0.07
54	A0A9L0ISH8	ZAR1	15600000	0.07
55	A0A8C4PT90	C1QL3	14700000	0.06
56	A0A8C4LJP2	ARID1A	14400000	0.06
57	A0A9L0K9S7	SCARA3	14100000	0.06
58	A0A8C4PRR0	CDK13	14000000	0.06
59	A0A9L0IXL3	EMID1	12900000	0.05
60	A0A8C4LX95	BSN	12800000	0.05
61	A0A9L0IHC8	OLFML2A	11900000	0.05
62	A0A8C4LKJ3	WDR33	11700000	0.05
63	A0A8C4MGK3	PGRMC2	10900000	0.05
64	A0A9L0K6I9		9600000	0.04

65	A0A8C4LJ11	HSPG2	9600000	0.04
66	A0A8C4PJ55	MARK1	8850000	0.04
67	A0A9L0K1N1	LUM	8810000	0.04
68	A0A8C4M698	PCF11	8790000	0.04
69	A0A9L0I4A2	PLXNB3	8770000	0.04
70	A0A8C4L9W7	PDPK1	8140000	0.03
71	A0A8C4KWX5	CBFA2T3	8060000	0.03
72	A0A9L0J3E7	LRRC24	8040000	0.03
73	A0A9L0IQQ6	SGIP1	7790000	0.03
74	A0A8C4L3F5	GREB1L	7550000	0.03
75	A0A8C4MSV2	SHANK3	7400000	0.03
76	A0A9L0IWR9	LOC106832448	6970000	0.03
77	A0A9L0J0L7	GPC2	6330000	0.03
78	A0A8C4M3X4	GLDN	5500000	0.02
79	A0A9L0JED9	LOC106839384	5440000	0.02
80	A0A8C4MDN4	NYAP1	5310000	0.02
81	A0A8C4MM08	SFTPD	5080000	0.02
82	A0A9L0JA29	BAP1	4790000	0.02
83	A0A9L0IS07	RINL	4700000	0.02
84	A0A8C4PHV1	SMARCC2	4360000	0.02
85	A0A8C4LE70	NTN3	4080000	0.02
86	A0A8C4LW45	PLEKHH3	3840000	0.02
87	A0A8C4MEB9	FBLN2	3670000	0.02
88	A0A8C4M542	GLIS1	3660000	0.02
89	A0A9L0J9I7	C1QTNF12	2930000	0.01
90	A0A8C4L4F9	ERFL	2900000	0.01
91	A0A9L0KHM2	ASH2L	2460000	0.01
92	A0A9L0JNM8	ERFE	2400000	0.01
93	A0A8C4MTI7	PPP1R10	2360000	0.01
94	A0A9L0JDK3	SCML2	2320000	0.01
95	A0A9L0J8P8	KDELR3	2270000	0.01
96	A0A8C4PJZ1	MED25	2190000	0.01
97	A0A8C4LKM8	TUBGCP5	2170000	0.01
98	A0A8C4LCX6	DNAH3	2140000	0.01
99	A0A8C4MI99	SNAPC4	1820000	0.01
100	A0A9L0JMN3	ANXA11	1810000	0.01
101	A0A8C4LG41	AGAP2	1710000	0.01
102	A0A9L0JY55	PRR36	1650000	0.01
103	A0A9L0J8U5	RBCK1	1640000	0.01
104	A0A9L0IPR1	PRR22	1630000	0.01
105	A0A8C4M322	EIF4G3	1620000	0.01
106	A0A8C4M8A9	FBRS	1570000	0.01
107	A0A8C4L9Y3	KIF23	1540000	0.01

108	A0A8C4MLS4	DCN	1530000	0.01
109	A0A8C4M3I8	OGN	1420000	0.01
110	A0A8C4LY43	GRM5	1370000	0.01
111	A0A8C4PNV9	WT1	1300000	0.01
112	A0A8C4KR55	NUDT16L1	1300000	0.01
113	A0A8C4L068	C1QTNF2	1300000	0.01
114	A0A8C4PGZ2	SRF	1210000	0.01
115	A0A9L0K0L1	TMEM271	1190000	0.01
116	A0A8C4MXG2	SF3A2	1160000	0.00
117	A0A8C4L9C8	CDC42BPB	1160000	0.00
118	A0A8C4KYX8	CHPF2	1160000	0.00
119	A0A9L0IWC9	SLC25A3	1110000	0.00
120	A0A9L0JL72	PAX9	1090000	0.00
121	A0A9L0KFF3	RHOQ	1040000	0.00
122	A0A8C4LGQ6	SMARCA4	900000	0.00
123	A0A9L0IQK3	STXBP4	895000	0.00
124	A0A9L0JFK2	HOXD1	857000	0.00
125	A0A9L0IJ82	LOC106836073	843000	0.00
126	A0A8C4MK39	CCER1	687000	0.00
127	A0A8C4M343	DDX4	667000	0.00
128	A0A8C4MI48	GATA4	651000	0.00
129	A0A9L0IK10	SLITRK1	650000	0.00
130	A0A9L0IEP0	GGT6	647000	0.00
131	A0A8C4L7I8	C1QL2	632000	0.00
132	A0A8C4N139	CSMD3	628000	0.00
133	A0A8C4LZK2	PRELP	591000	0.00
134	A0A9L0I5Z7	HOXD9	564000	0.00
135	A0A8C4MQ19	MDC1	534000	0.00
136	A0A9L0IDT9	LRRC15	518000	0.00
137	A0A9L0K2E6	UBQLN2	467000	0.00
138	A0A8C4ML47	SH3BP1	456000	0.00
139	A0A9L0KGD8	BEAN1	435000	0.00
140	A0A9L0IAD0	CTDP1	422000	0.00
141	A0A8C4L9T6	FAM83C	408000	0.00
142	A0A9L0IBR6	SYNC	385000	0.00
143	A0A9L0J115	TOP2B	382000	0.00
144	A0A8C4MYS4	SEMA6B	362000	0.00
145	A0A8C4MTE7	LHFPL5	342000	0.00
146	A0A8C4LDH4	RYR2	328000	0.00
147	A0A8C4MBT5	ATP2A1	317000	0.00
148	A0A8C4LQL5	TMEM249	289000	0.00
149	A0A8C4MXU7	ARL1	288000	0.00
150	A0A9L0IPB9	RP1L1	254000	0.00

151	A0A8C4L6L9	NEO1	247000	0.00
152	A0A8C4PRE0	ALB	241000	0.00
153	A0A8C4MT04	RBM27	228000	0.00
154	A0A9L0IB39	CLEC20A	222000	0.00
155	A0A9L0IDB4	SPAG5	191000	0.00
156	A0A8C4PLM4	C1QL1	181000	0.00
157	A0A8C4LGB9	ALS2CL	179000	0.00
158	A0A9L0JQS9	LOC106843847	161000	0.00
159	A0A8C4MSV8	ARSA	151000	0.00
160	A0A8C4KUA4	PRPF40B	141000	0.00
161	A0A9L0K6M6	LOC106824939	135000	0.00
162	A0A8C4MLZ7	EP400	132000	0.00
163	A0A9L0JSR3	DCHS1	130000	0.00
164	A0A8C4LGX8	COLQ	129000	0.00
165	A0A9L0J224	SYNPO2L	120000	0.00
166	A0A8C4PIR3	GLB1	105000	0.00
167	A0A9L0IA09	CGGBP1	102000	0.00
168	A0A8C4LLA1	AHDC1	101000	0.00
169	A0A8C4MP04	ADAMTS17	56400	0.00
170	A0A8C4M3X8	ZDHHC7	47500	0.00
171	A0A9L0JJR3	MIEF1	33300	0.00
172	A0A9L0JY56	EDA	27400	0.00
173	A0A8C4LER9	TRIM33	12400	0.00

[1] Accession numbers refer to the UniProt database. -10lg*P* denotes the protein identification confidence score; relative abundance was normalized from the peptide-spectrum match (PSM) area.
